# Correspondence

**Published:** 1914-03

**Authors:** J. W. Stewart

**Affiliations:** Ithaca, N. Y.


### CORRESPONDENCE.

*This department is intended for the presentation of news and views not coming within the scope of other departments, and particularly to afford opportunity for discussion and criticism of views elsewhere expressed.*

---

Ithaca, N. Y., February 10, 1914.

Editor BUFFALO MEDICAL JOURNAL:

I would be greatly indebted for the names of any reputable physicians of this section of the country who have reported successful use of Hypnotic Suggestion in the treatment of disease.

J. W. STEWART.

